# Influence of Section Thickness on the Accuracy and Specificity of Histometric Parameters Using Confocal Laser Scanning Microscopy in a Canine Model of Experimental Peri-Implantitis—A Proof of Concept

**DOI:** 10.3390/jcm12072462

**Published:** 2023-03-23

**Authors:** Lucia-Camelia Boldeanu, Aurel Popa-Wagner, Marius Boariu, Stefan-Ioan Stratul, Darian Rusu, Octavia Vela, Alexandra Roman, Petra Surlin, Georgios Kardaras, Salvatore Chinnici, Adrian Vaduva

**Affiliations:** 1 Department of Periodontology, Faculty of Dental Medicine, Anton Sculean Research Center for Periodontal and Peri-Implant Diseases, “Victor Babes” University of Medicine and Pharmacy, 300041 Timisoara, Romania; 2 Department of Neurology, Chair of Vascular Neurology and Dementia, University Hospital Essen, 45147 Essen, Germany; 3 Center for Experimental and Clinical Medicine, University of Medicine and Pharmacy Craiova, 200349 Craiova, Romania; 4 Department of Endodontics, Faculty of Dental Medicine, TADERP Research Center, “Victor Babes” University of Medicine and Pharmacy, 300041 Timisoara, Romania; 5 Applicative Periodontal Regeneration Research Unit, Department of Peridontology, Faculty of Dental Medicine, Iuliu Hatieganu University of Medicine and Pharmacy, 400012 Cluj Napoca, Romania; 6 Department of Periodontology, Faculty of Dental Medicine, University of Medicine and Pharmacy, 200349 Craiova, Romania; 7 Department of Pathology, Faculty of Medicine, ANAPATMOL Research Center, “Victor Babes” University of Medicine and Pharmacy, 300041 Timisoara, Romania; 8 County Emergency Hospital, L. Rebreanu Street, nr. 156, 300723 Timisoara, Romania

**Keywords:** animal study, dental implant, ligature induced, peri-implantitis, histomorphometry, confocal laser scanning microscopy, autofluorescence

## Abstract

Objectives: Tissue breakdown was assessed by confocal laser scanning microscopy (CLSM) using autofluorescence around implants with ligatures, on a dog hemimandible. Influence of section thickness on the accuracy of histometrical observations was also evaluated, in comparison with thin sections in light microscopy. Material and Methods: Three months after tooth extraction, implants were placed. Two months after abutment placement, ligatures were placed with no plaque control. 11 months post-implantation, the animal was sacrificed. Undecalcified thin (30 µm) sections were cut, stained and evaluated by light microscopy to be used as a reference. Additional sections were performed, so that another pair of unstained thick sections resulted (250–300 µm). Tissue loss was assessed using histomorphometric parameters under CLSM and was compared to the light microscopy reference ones. Results: Morphometry confirmed tissue loss more pronounced on the “thick” and quick sections, when compared to the time-consuming and technique-sensitive “thin” ones. Conclusions: Within the limits of the present study, the adequacy of histometrical observations under CLSM reveal commensurable information about soft-tissue-bone-implant details, when compared to traditional light microscopy histological protocols. The CLSM investigation may seem demanding, yet the richness of data acquired may justify this approach, provided seatbacks caused by improper manipulation of “thick” sections are avoided.

## 1. Introduction

Peri-implantitis is considered the most common biological complication in implant therapy [1,2,3,4,5]. Clinically, the features of this infectious disease include bleeding on probing, while radiographic and histopathological analysis reveal loss of supporting marginal bone and severe inflammation of the tissues that surround the implant [1,6,7]. The new Classification for Periodontal and Peri-implant Diseases and Conditions (2018), defines peri-implantitis as a plaque-associated pathologic condition occurring in the tissue around dental implants, characterized by inflammation in the peri-implant mucosa and subsequent progressive loss of supporting bone [8]. Although imagistic data during the loss of bone support in humans could be very easy obtained [9], more histopathological information about peri-implantitis can only be obtained from soft and hard tissue biopsies, and they are regulated by technical and ethical directives related to the sampling of tissues from humans [10]. The animal model, in dogs especially, was believed so far as a source of adequate and comprehensive information regarding the tissue reactions in experimentally induced peri-implant diseases [10,11,12,13].

Different protocols have been suggested in the processing of the samples containing hybrid bone-implant specimens. The original protocol of Donath (1982) for ground sections of undecalcified bone tissue with implants in situ facilitates the production of increasingly thinner sections (5–10 µm), assuming to provide more accurate histological information [14]. Since it has been imagined, variations of this classical sawing-grinding protocol have been developed, which led to obtaining sections of 20 µm [1,15,16,17,18,19], 30 µm [20,21,22], 40 µm [23,24,25], 100 µm [26,27,28] and 150 µm [29], for various research purposes.

Previous own research indicated good results in the histological observation of secondary non-demineralized bone-implant wet samples of greater thickness (150–200 µm), analyzed extemporaneous immediately after sectioning, without grinding and fixation on glass slides, using both standard and fluorescent staining [30]. These thicker sections are fast to obtain, are cheaper to create since the slide fixation is no longer required, and easier to manipulate under the microscope. So far, there is no investigation at what extent such thicker sections (>200 µm) could provide relevant and reliable histological results, when compared to thin sections of non-demineralized specimens of peri-implantitis, as per traditional histological protocols. In the above-mentioned studies, the thickness of the sections seemed to be dictated by individual choices of the authors, based on certain aims of the research, rather than on a clear correlation between the thickness of the sections and the aimed histological findings. 

Developed relatively recently, the confocal laser scanning microscope (CLSM) focuses only on a single focal plane, and the unfocused plane remains unvisualized. The technique utilizes a laser beam, which penetrates tissue to a depth of 300–500 μm and thus reflects images beneath the surface of a specimen [31,32,33]. In the past, the traditional fluorescent microscopes excited the whole thickness of the sample, resulting in saturated, blurry images and sometimes visualizing false colocalization images [34]. Since the CLSM excites a single focal plane, this technique is suitable for quantification and visualizing “true” images. CLSM allows modulation of the intensity of the signal and the microscope can pick up the weakest signals expressed in the sections as autofluorescence, especially useful for the study of bone destructive processes [35]. 

The advantages of CLSM are its ability to view the structures within a specimen or cell and the fact that it can be used with various specimens, including unfixed, wet specimens. CLSM has been already used for viewing the structures at the implant-tissue interface, such as unmineralized bone matrix or mineralized bone [31,32,33]. With modern computer technology and software, a series of optical sections obtained using CLSM can be recombined to create a 3D image of a cell or structure even when using multiple labeling techniques [36].

CLSM has been occasionally used for the study of bone regeneration following experimental ligature-induced peri-implantitis, on cuting-grinding thin sections, with a focus on the influence of the rate of progression of the lesion on the outcomes of the regenerative therapy [37]. There is only a human case report of a bisphosphonates-related osteonecrosis around a dental implant that has been investigated using histology and CLSM [38]. However, the literature does not mention studies using CLSM that evaluate primarily the tissue loss around experimentally-induced peri-implantitis on non-decalcified sections. As data obtained under CLSM are not influenced by the thickness of the section, a simplification of the classic, costly, time-consuming and technique-sensitive cutting-grinding protocol may be of interest in the histomorphometrical study of peri-implantitis.

The aim of this pilot study was to determine whether the standard histological and histomorphometrical landmarks are available on thick vs. thin sections of non-demineralized sections of bone-implant-soft tissue specimens in experimentally induced peri-implantitis in dogs, using the CLSM. The influence of specimen thickness on the accuracy of the measurements of distances and angles of tissues degraded by the experimental peri-implantitis was also evaluated. For comparison, measurements in previously obtained thin sections (30 µm) for light microscopy were used.

## 2. Materials and Methods

This study was conducted in a single animal, to minimize the number of experimental animals to be sacrificed while obtaining a maximum of data [39]. 

### 2.1. Animal Protocol

The protocol of the whole experiment was approved by the Ethical Commission of Scientific Research of Victor Babes University of Medicine and Pharmacy Timisoara (approval Nr. 06-16/2019). The experiment was finalized in December 2019. Dogs have a long history in periodontitis and more recently peri-implantitis research and remain the researcher’s animal of choice [40]. Until now they have been almost exclusively used as an animal model for the purpose to mimic the human naturally occurring peri-implantitis with respect to onset and progress [41]. Moreover, the predisposition to gingivitis/peri-implant mucositis and subsequent periodontitis makes the dog model suitable for studies for biofilm deposition and occurrence of peri-implantitis [42]. The animal protocol was described in detail in a previous published paper (Boldeanu et al. 2022) [43]. In this study, one ten-years old adult half-breed dog, with a fully erupted permanent dentition (male, body weight 20 kg) was used. The dog was housed under good general conditions in a single kennel with indoor and outdoor areas. The room temperature range was approximately 18 °C, with a humidity above 30%. It was fed once a day using granulated dog food and water ad libitum. The maxilla was intact, without any general occlusal trauma, oral viral or fungal lesions. Clinical examination determined that the animal was in good general health, with no systemic involvement. All procedures were carried out under the supervision of two veterinary surgeons, one of them in charge of the anesthetic procedures. Healing was evaluated weekly, and the dog was fed a soft diet for 14 days after the sutures were removed.

### 2.2. Surgical Procedures

All operative procedures were carried out under general anesthesia (intravenous Diazepam 0.5%, 0.4 mg/kg I.V. and Ketamine 10%, 10 mg/kg I.V., endo-tracheal intubation 2–5% isoflurane gas). To maintain hydration, the animal received a constant-rate infusion of Ringer’s solution while anesthetized.

**Figure 1:**
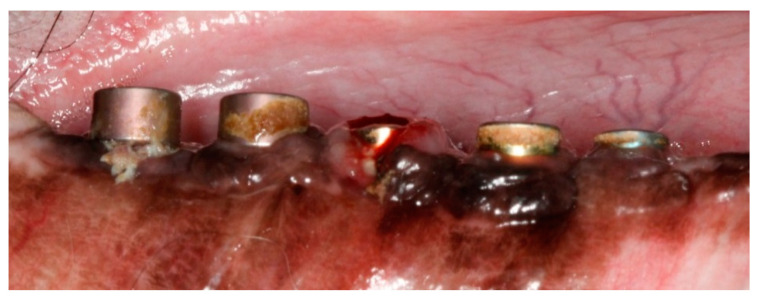
Post-operative image at 9-month post-implantation. Note the ligatures placed in implants 1, 3 and 5, and the subsequent local inflammation.

The premolars and the first molar were extracted after reflection of full thickness mucoperiosteal flaps from the posterior mandibular region on the right side. Three months later, five 8-mm screw-shape endosseous titanium implants were inserted in the edentulous regions using standard instruments and surgical techniques. Implants were exposed 3 months post-implantation and their healing abutments were connected. No oral hygiene regimen was administrated during this period, so that the peri-implant inflammation could initiate spontaneously. Five months post-implantation (two months post implant exposure), to accelerate the progression of the initial lesions, alternative cotton ligatures were placed according to the method described by Lindhe et al. [44] in a submarginal position around the neck of implant (Figure 1). The animal was then fed a soft diet to induce plaque accumulation and to provoke peri-implant inflammation and loss of bone.

### 2.3. Specimen Preparation

At the end of the experiment, 8 months after abutments were installed, the dog was sacrificed under general anesthesia (sodium pentobarbital: 200 mg/kg i.v.). The jaw was sectioned at the midline, a segment of mandible including the implants and ligatures in situ (8 cm × 4 cm) was separated and fixated in 10% neutral buffered formalin until laboratory processing. 

### 2.4. Histological Preparation of the Specimens

From the entire bone segment including all five implants, separate sub-segments were prepared by trimming down to the zone of interest, as follows: cuts were carried out in buccal-lingual direction resulting three separate segments. First segment with implants No. 1 and No. 2, second segment containing implants No. 3 and 4, and the third segment containing implant No. 5. Specimens were dehydrated in increasing concentrations of ethanol (30%, 50%, 60%, 70%, 80%, 90%, 2× 96%, 99%) followed by 2× xylene at room temperature (20 °C), under slight shaking, in a slow procedure, over 14 days. Technovit 9100 (Kulzer GmbH, Hanau, Germany) embedding resin was used for the infiltration and embedding steps, which were performed at temperatures between minus 2 °C and −12 °C, allowing polymerization, using a vacuum pump. Sections were prepared using the cutting/grinding method described by Donath & Breuner [14] on a cutting/grinding system (Exakt, Norderstedt, Germany). The polymerized resin blocks containing the embedded sample were trimmed and transferred to glass slides. Consecutive numbers and letters were attributed to each implant and subsequent sections, in order to identify them. A cut was performed in buccal-lingual direction through the middle of each implant, in the long axis. For each implant, one section was carried out distally from the initial mid-section, while another section was carried out mesially, both of them in the long axis, so that two relatively equal central sections resulted. Each of these two sections were thinned and mechanically micro-polished, stepwise, using a Micro grinder machine (Exakt 400 CS, Norderstedt, Germany) reaching the final thickness of approximately 30 µm, and were used in a previously published research (Boldeanu et al. 2022) [43]. Additional sections were performed mesially and distally from the thin ones, so that another pair of sections resulted. These sections measured between 250 and 300 µm and were not thinned or mechanically micro-polished. Finally, the thick sections were not dehydrated and were not cover-slipped with mounting medium. Between examinations, to prevent curling, the thick sections were kept between flat surfaces by placing a heavy flat object on top. During examinations, these sections were manipulated by hand using fine tweezers. 

Sections were evaluated on an Olympus Fluoview FV1000 confocal microscope (Olympus, Tokyo, Japan), using a 10× NA:0.40 UPLSAPO objective, 2 µs/pixel sampling speed, confocal aperture of 80 µm Z-stacks of 2048 × 2048 images were acquired and stitched using the grid/collection stitching plugin in ImageJ [45]. The stitched z-stacks were visualized in Imaris v7.4.2 (Bitplane AG, Belfast, UK) and measurements were performed by one experienced investigator (AV). Prior to the start of the analyses, a calibration procedure was initiated for the investigator and revealed that repeated measurements of different histological sections were similar at >95% level.

The “golden standard” light microscopy (LM) histomorphometry was used in the present research as a reference. Means and standard deviations used in this study as reference were calculated from histomorphometric measurements presented in a previous paper (Boldeanu et al. 2022) [43]. The stained thin sections were scanned using the microscope slide scanner Leica Aperio^®^ AT2 (Leica Biosystems, Wetzlar, Germany), under the 40× objective. 

### 2.5. Histomorphometrical Landmarks, Distances and Angles

**Figure 2:**
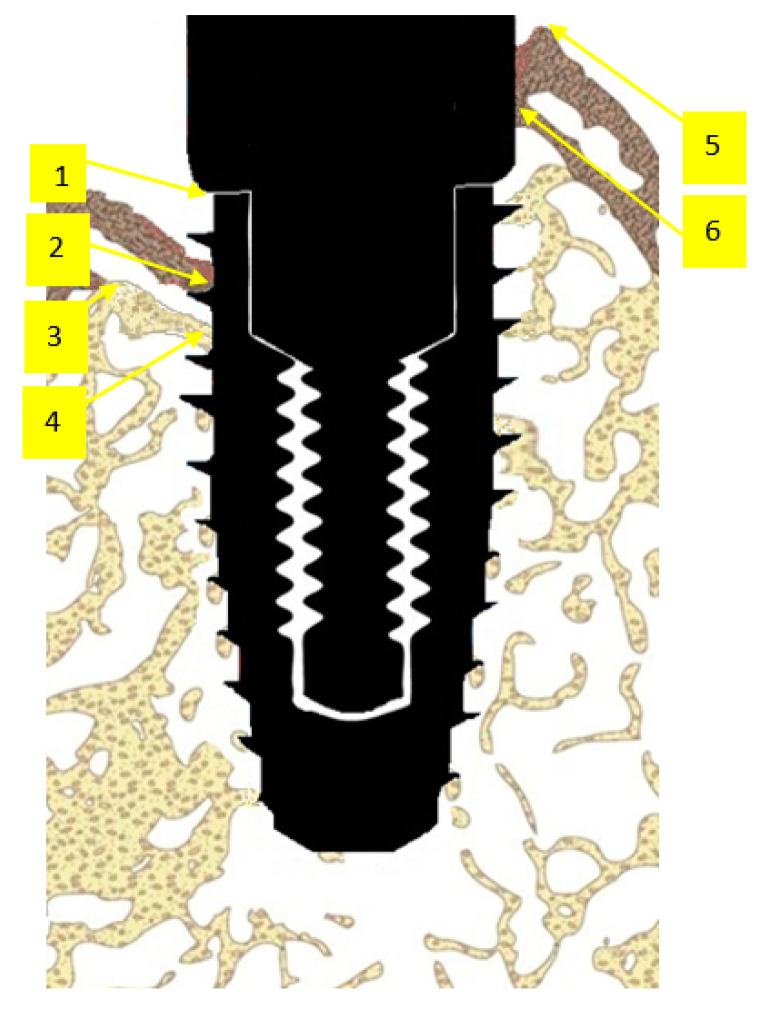
Histomorphometric landmarks identified on both buccal and lingual aspects in each implant: 1—implant shoulder (IS); 2—implant surface at the level of the bone crest (imp); 3—bone crest, defined as the most coronal point of the bone (BC); 4—bottom of the bone defect (BD); 5—margin of the peri-implant mucosa (PM); 6—the most apical extension of the submarginal biofilm that was interposed between the implant and the pocket epithelium of the peri-implant mucosa (aPlaque).

Histomorphometric landmarks that were identified on the “thin” (30 µm) and “thick” (250–300 µm) sections on the buccal and lingual aspects of each implant are presented on Figure 2. Linear measurements on both aspects of each implant are presented in Table 1.

### 2.6. Data Analysis

As the sample size in this proof-of-concept study was small, and the data needed to be more cohesive, a reliable statistical analysis could not be performed. Since data don’t follow a normal distribution, means and standard deviations were used to describe the data set. They were calculated for all implants, for horizontal, vertical and angular measurements of each implant, on the buccal and lingual aspects. Where landmarks were not visible on all sections, and subsequent measurements were missing, means were calculated in relation with the number of available sections. 

## 3. Results

During the experiment, no implant was lost. Heavy biofilm accumulation was observed around all healing abutments, the peri-implant tissues became inflamed, bleeding was present, and one implant exhibited suppuration. During the histological processing and manipulation of the “thick” sections, some minute areas, including both tissue and metallic fragments, were lost, which precluded the observation/recognition of several landmarks. In order to be able to obtain a full scan to include the landmarks mentioned above, between 10 and 21 Z-stacks image sets were acquired per specimen, including up to 118 optical scanning planes, that were later used for 3D reconstructions (Videofiles No. 1, 2). The average penetration depth in the thick sections was 90 µm, while the thin sections were completely evaluated in thickness.

### 3.1. Histological Observations

#### 3.1.1. Qualitative Comparison between the Light Microscopy, CLSM Thin, and CLSM Thick Sections

**Figure 3:**
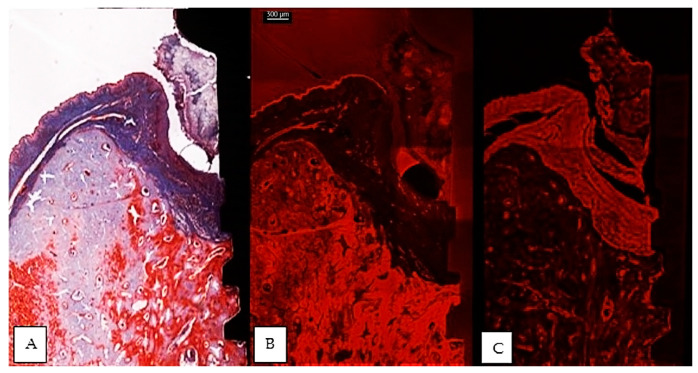
Comparative images of the same implant, between a thin section in light microscopy, Masson Goldner Anilin blue stain (**A**), same thin section in CLSM (**B**) and a thick section in CLSM (**C**). Morphological preservation of tissular integrity is better seen in the thick section. (implant #5, buccal aspect, bar measure for all three images is 300 µm).

**Figure 4:**
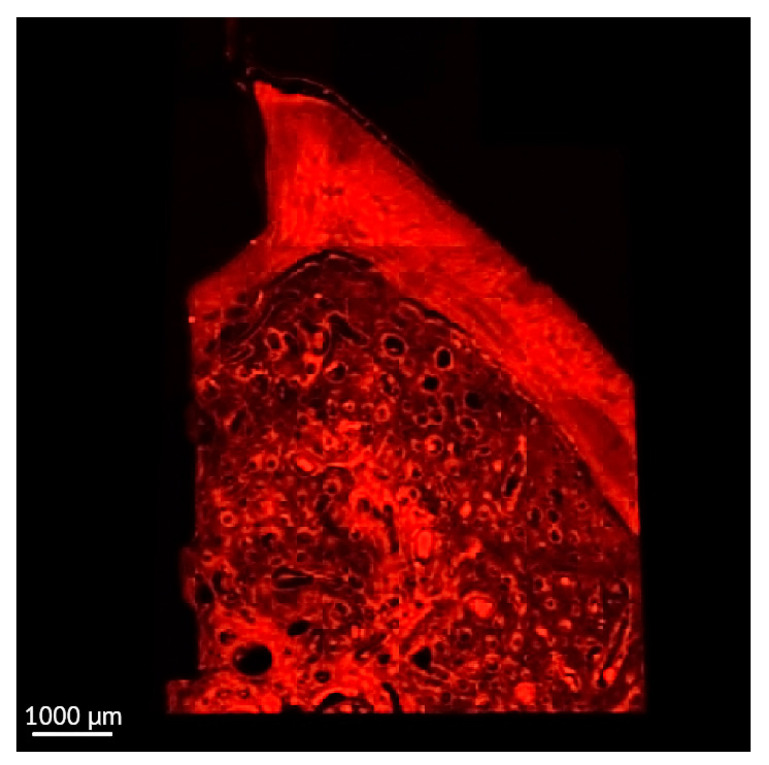
Soft tissue and bone in a thick section of a non-ligated implant. Bone is in contact with the implant surface. Soft tissue is in contact with the healing abutment. (implant #2, oral aspect; bar, 1000 µm).

**Figure 5:**
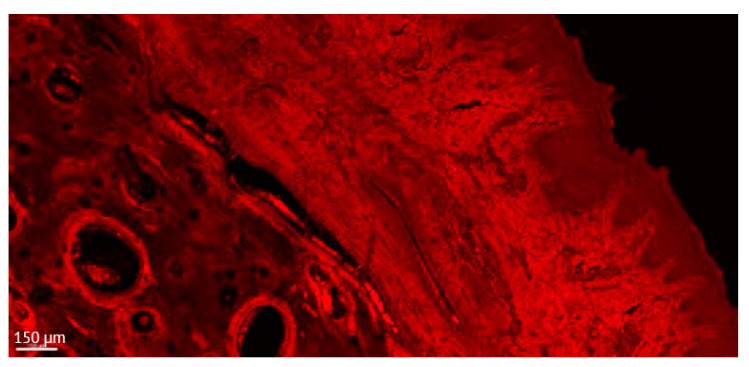
Detailed aspect of the soft tissue and bone in a thick section of a non-ligated implant. Note the presence of acanthosis of the covering epithelium. ICT is present in contact with bone and implant, promoting an active bone resorption. (implant #2, oral aspect; bar, 150 µm).

**Figure 6:**
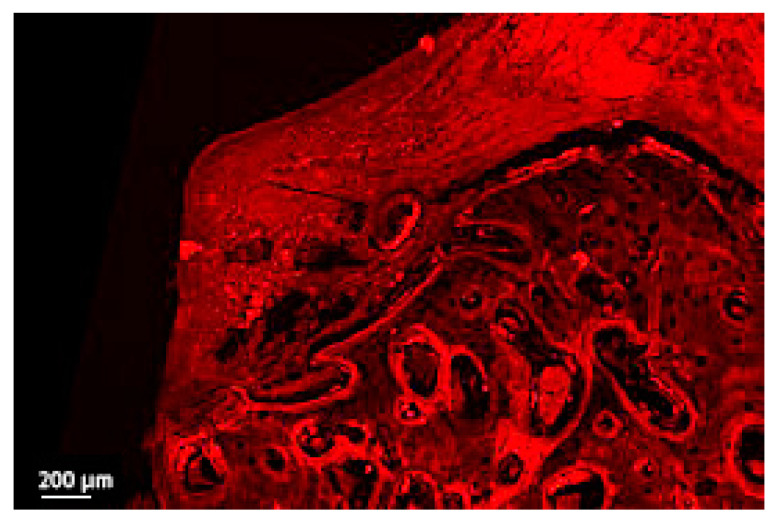
Detail of the soft tissue bone in a thick section of a non-ligated implant. Note the ulcerated buccal pocket epithelium, the interrupted epithelial lining, the infiltration of inflammatory cells and the disruption of collagen network (implant #2, oral aspect; bar, 200 µm).

**Figure 7:**
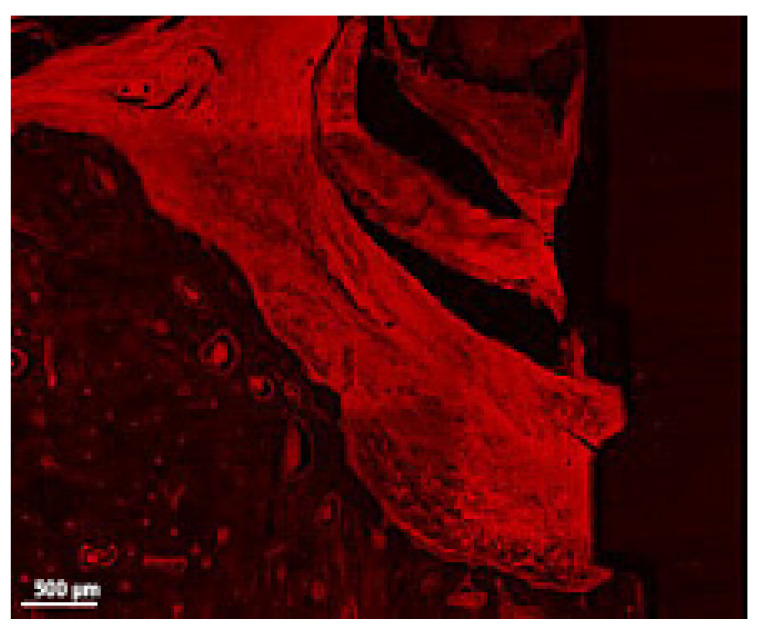
Detail of the soft tissue and bone in a thick section of a ligated implant. Note the presence of ligatures in the upper central region of the image, the highly inflamed soft tissue in contact with bone and implant surface, the minimal presence of Howship lacunae and the congested venules (implant #5, buccal aspect, bar, 500 µm).

The usual light microscopy technique using pentachrome stains revealed good tissue morphology preservation; however, the opacity of the metallic implant made it difficult on some of the specimens to properly identify the implant shoulder. The CLSM thin sections were easier to scan, having a flatter surface, yet the grinding to a thin section led to minor tissue cracks in the bone component (Figure 3A,B). Furthermore, the use of mounting medium and coverslip on the thin sections induced an additional decrease in contrast, altering the quality of soft tissue morphology in the thin sections (Figure 3A,B). In both thin and thick sections, the implant shoulder was easily identifiable, except in the thick sections where the implant was lost during histological processing. The peri-implant sulcus is shallower in non-ligated specimens than in ligated ones, the bone is in contact with the implant surface and the soft tissue is in contact with the healing abutment (Figure 4). Infiltrated connective tissue (ICT) was present in contact with bone and implant (Figure 5). However, it is important to mention that the cellularity of the ICT was not visible under CLSM; therefore, LM seemed better suited for the assessment of the ICT. Epithelial lining integrity was broken, more pronounced on the oral aspect, making the identification of landmarks like aJE extremely difficult (Figure 6). This comes not as a surprise under the severe inflammation circumstances found in peri-implantitis, as this landmark is poorly identifiable even on LM. In these areas of integrity loss, the highly inflamed soft tissue was identified in direct contact with the bone and implant (Figure 7). Regarding the soft tissues, the epithelial covering is clearly identifiable on both types of evaluation (LM and CLSM).

#### 3.1.2. 3D Reconstruction Analysis

The 3D reconstruction of the “thick” sections resulted in impressive images, based on tissue autofluorescence. The bone was clearly visible in contact with the implant surface. The highly cellular, inflamed ICT was present in contact with the bone and the implant, and also with the healing abutment, creating a deep soft-tissue pocket, as seen in the non-ligated implant #4, on the buccal aspect (VideoFile No. 1). However, in ligated implant #5, on the oral aspect (VideoFile No. 2), the ICT is in contact with the implant surface and apparently progresses into the bone marrow. The peri-implant sulcus is deep, and the intimate contact between the soft tissues and the implant is below the BC level, describing a crater-like bone defect.

### 3.2. Histometric Findings

The results from the histometric measurements are reported in Table 2. For comparison, means and standard deviations were calculated from histomorphometric measurements in thin sections under light microscopy, as presented in a previous paper (Boldeanu et al. 2022) [43].

## 4. Discussion

Traditionally, the commonly employed histologic analysis techniques for bone and teeth involve decalcification [46]. The preparation of thin undecalcified bone sections by a rapid manual method was attempted by Frost (1958) [47]. Modern histologic protocols to obtain undecalcified ground tissue samples were mentioned in the early 1970s, whenDelling et al. developed a simplified methacrylate embedding method for thin undecalcified bone sections (Delling 1970) [48] and performed the embedding of iliac crest bone biopsies in methylmethacrylate (1972) [49]. Thin sections of undecalcified bone and surrounding soft tissues were obtained by Kalkweit (1971) [50], while developing an improved method that enabled methacrylate-embedded bone corticalis to be cut with a fast running saw into sections of 20–40 μm thickness, without loss [51].

Ground sections of undecalcified bone tissue with implants in situ were performed with various equipment. Depending on the utilized technique, various qualities of the sections resulted, which may be of importance when performing histomorphometric analysis of the bone tissue surrounding implants [52]. The Donath protocol, designed for hybrid bone-implant samples, facilitated the creation of increasingly thinner sections, believed to provide more accurate histological data with respect to cellular and fibrillar elements of the targeted areas (regions of interest) [14].

Various section thicknesses seemed to be dictated by the individual choices of the authors, based on particular goals of the research, rather than on a clear correlation between the thickness and the aimed histological findings. In general, the papers provide no rationale for the choice of a particular thickness. Moreover, the histological literature comparing the same elements in sections of various thicknesses is very scarce. A study from 1995 investigated the bone-to-metal contact on sections of 10, 30, 50 and 100 µm, concluding that ground sections that are over 30 µm may result in an overestimation of the “true” bony contacts. The authors mentioned that quite often rather thick ground sections (up to 500 µm thick) are being investigated but concluded that theoretical calculations of the bone-to-metal „direct” contact on thin (10 µm) sections in comparison to thicker ones, have shown that misinterpretations of the bone estimation are more frequent with increasing section thickness [52]. In this respect, our proposed method of CLSM examination of thick sections comes to fill the gap, as it allows the evaluation of individual optical sectioning planes and full 3D reconstruction of the bone-implant contact surface. 

Thicker sections have been occasionally critically assessed by several authors. In thin sections (10 µm), the bone did not always come into direct contact with the implant, and an amorphous or connective tissue zone with or without mineralized matrix was interposed between the implant and highly mineralized mature bone or newly formed lamellar bone, while in thicker ground sections bone appeared to form direct contact with the implant [53]. It was also noted in osseointegration experiments that, when analyzing thick sections (100–500 µm), similar bony contacts may be observed, irrespective of the implant biomaterial. Furthermore, it is not unusual to find quantitative results performed on sections that are between 70 and 100 µm thick [52]. The benefits of thicker sections of hybrid specimens have been also correlated with different grinding rates for titanium and bone, as thinning of the specimens results in different thicknesses on the same section which possibly leads to edging effects at the interface. Moreover, tissue might be separated from the implant surface and, additionally, may be destroyed throughout the grinding procedure, as noted in our study on the “thick” sections.

In addition, the significant additional expense involved in the preparation of analyzable stained histological specimens and their susceptibility to errors need to be taken into account [54]. These findings must be considered critically, especially when preparing thinner stained sections. On the other hand, despite an increasing accuracy of the potentially visible histological details, very thin sections proved sometimes disadvantageous with respect to the handling of the specimens, the complexity of the fixation procedures and the duration of the grinding, and the creation of artifacts that could jeopardize the quality of the observations. In sections that are reduced to approx. 30 μm, scratches on the surface, extrusion of the implanted material, cracks, areas of sheer tissue loss, and uneven thicknesses may result in poor cellular detail, as noted in our study on the “thin” sections. [31] (Figure 3A,B). Many researchers agree that great technical skills are needed in obtaining satisfactory thin sections by maintaining parallelism during the grinding procedures. It is also known that during grinding the hard metal normally is depressed more on the sandpaper than the adjacent softer tissue, and this causes the surrounding tissue to abrade faster than the metal. Yet, the undecalcifed technique has been used widely for oral implants [55,56,57,58], despite the fact that processing artifacts in the histological very thin sections are still common [55].

Although great care is taken to preserve the implant within the ground section, it is not possible to determine whether the interface is preserved well during the cutting and grinding processes. Chai et al. observed that titanium discs were dislodged, although they were intact after cutting with a diamond band saw. In our research, this happened more often in the off-center “thick” sections, pointing to the advantages of performing “thick” sections in the first place. Indeed, while examining the interface surface of the resin block using a confocal laser scanning microscope, the aforementioned authors noted that the interface was irregular, suggesting that the force of cutting may have dragged the tissue or disturbed the interface [59].

Early authors noted that a thickness of 80 to 100 µm (taking into account about 20 µm for the layer of glue or cement) should be sufficient for all the methods of microscopic examination of non-demineralized specimens and preparing thinner sections down to a thickness of 5 to 10 µm is, in most cases, unnecessary, requiring machine grinding and being associated with more risk of failure. However, they mentioned that thin sections (0.5 to 5 µm thickness) can provide additional information about cellular details of the implant-tissue interface [60]. In our study, such information was provided under CSLM by the “thick” sections (Figure 4).

Human bones exhibit fluorescence, typically induced by natural antibiotics that are absorbed by collagen, and provide secondary, exogenous fluorophores. However, primary natural fluorescence (or autofluorescence) caused by enigmatic endogenous fluorophores is also present as a micro—phenomenon, whose nature is still obscure [61]. Collagen fibers in unmineralized osteoid appear to have pronounced autofluorescence [31]. While experimentally injected fluorescent substances are used for the study of hard tissue mineralization and bone aposition/regeneration, autofluorescence seems useful for the study of bone destructive processes, like in the present study of experimental peri-implantitis.

Regarding the soft peri-implant tissue, some authors stated that, like the SEM technique, CLSM allows visualization in situ of the implant-tissue interface even without embedding or cutting [59]. Similar to our study design, other authors combined different microscopic techniques for the analysis of an oral implant. For instance, in a study from 2001, the tissue was triple-labeled fluorescently for the adhesion molecules involved in the interface, then examined under CLSM [62]. Although this method allowed inspection of fluorescently labeled components, its limitation was that it couldn’t obtain high-resolution images of ultrastructural features such as hemidesmosomes. On the other hand, in a very recent study analyzing the perpendicular attachment of connective tissues, with confocal laser scanning microscopy it was demonstrated that the fibroblasts had attached and proliferated over the entire micropore surface, forming a prolific cell sheet and also, 3D imaging (confocal z-stacking) confirmed the migration of cells into the micropore structures [63]. In our study, where autofluorescence was employed, similar observations were made when recombining the “thick” sections in 3D (Videofile No. 1). The result was an increase in the quality of soft tissue images when compared to the “thin” sections. The latter seemed blurred by the use of mounting medium and coverslip.

Considering the hard tissues surrounding the implant, different points of interest defined by histological landmarks were used by authors according to the aim of their experiments. In our study, we used landmarks to asses the bone loss progression. However, not all points of interest (landmarks) were always visible, for various reasons [64]. Parameters that are relevant for assessing bone resorption and for defining the existence of a certain stage of peri-implantitis have been successfully acquired in our study (imp-BC, BC-BD, PM-BD, and the angle between IS-BD and BD-BC). When the implant is detached from the slide, because of accidental or incorrect histological processing of the specimens, the implant shoulder also disappears, thus landmarks like IS become not detectable (Figure 4). 

Our findings revealed minor differences between the mean distances under CLSM for both “thin” and “thick” sections, when compared to LM “thin” sections (Table 2). This is an expected observation, especially for the CLSM “thin” sections compared to the reference “thin” ones, where the implant shoulder (IS) was clearely visible. For example, mean IS-BC was 2.26 ± 0.95 on the buccal aspect and 0.34 ± 0.65 on the lingual aspect for the CLSM “thin” sections vs. 2.24 ± 1.11 on the buccal aspect and 0.57 ± 0.52 on the lingual aspect for the LM “thin” sections. 

A slightly overestimation of the peri-implant mucosa seems to appear in the CLSM sections when comparing PM-BD and PM-IS with the reference ones. The mean distance between PM-BD on the “thick” sections (4.12 ± 1.47 on the buccal aspect and 4.05 ± 0.70 on the lingual aspect) vs. the CLSM “thin” sections and the reference ones (2.77 ± 1.01 on the buccal aspect and 2.70 ± 1.38 on the lingual aspect, and 2.84 ± 1.12 on the buccal aspect and 3.01 ± 1.48 on the lingual aspect, respectively) would signify a deeper peri-implant pocket on the “thick” sections. This can be attributed to the fact that the “thick” sections contain supplementary soft-tissue, facilitating a more realistic peri-implant pocket measurement, in contrast to the “thin” section that can contain only a small section of the peri-implant mucosa. This also applies when comparing the mean results for aPlaque-BD on the “thick” sections (3.24 ± 0.28 on the buccal aspect and 2.39 ± 0.62 on the lingual aspect) vs. the CLSM “thin” sections and the reference ones (2.03 ± 0.58 on the buccal aspect and 1.49 ± 0.23 on the lingual aspect and 2.39 ± 0.55 on the buccal aspect and 2.21 ± 0.46 on the lingual aspect, respectively).

An interesting observation is the closely correlation between the mean results for the angle between IS-BD and BD-BC in the “thick” sections (75.6 ± 16.9 on the buccal aspect and 54.25 ± 16.13 on the lingual aspect) vs. the reference ones (77 ± 41.99 on the buccal aspect and 47 ± 11.13 on the lingual aspect), meaning that on the “thick” sections the architecture of the resorbed bone can be clearly distinguished.

In general, a good surface preparation is crucial for obtaining high-quality 3D imaging using a confocal microscope. A smooth and flat sample surface can minimize the number of scan planes required, which can reduce the computational resources needed for image processing. Additionally, a well-prepared sample can also reduce noise and artifacts in the final images, resulting in better image quality. Scanning in CLSM is time-consuming, especially when a laser scanning confocal microscopy and not a spinning disk confocal microscopy is used. Finally, CLSM requires image processing infrastructure with high computing power. Both this issues occurred in our study: as smooth, flat surfaces of the thick samples were scarce (e.g., implants No. 2, Figure 4, Figure 5 and Figure 6), the required number of scan planes in the other implants was high, leading to a higher time consumption. On the other hand, an increased number of scan planes results in spectacular 3D reconstructions, as provided by our study (Videofile No. 2).

There are several advantages of CLSM over LM, as a good method to identify both soft and hard tissue modifications, especially when analyzing thicker sections, that tend to appear opaque in LM: there are no laborious staining protocols because CLSM uses intrinsic fluorescence; the “thick” sections can be easily manipulated by hand; it can provide 3D reconstructions. Particular disadvantages of CLSM when compared to LM resulted from the observation of this study: the ICT, a relatively important area when describing the inflammation in peri-implantitis, cannot be identified neither characterized properly without supplementary fluorescent labelling (Figure 7), same for the limit of the squamous epithelium (aJE) that did not appear clearly visible on many sections (Figure 6).

A consensus for establishing an exact thickness for the section containing hybrid bone-implant non-demineralized tissue samples that fulfils both histological and histomorphometrical demands remains largely hard to obtain, due to different aims of the studies, to variations of the technologies and software used, and last but not least, to personal experience and affinities for some histological processing protocols. The major drawback of the present study represents the loss of tissues and of the implant during the histological process. Such shortcomings can be mitigated by a faultless embedding processing, a careful manipulation during the sectioning phase and handling with care between examinations by placing them between a plane surface with a heavy object on top to avoid curling. Another shortcoming is the number of implants inserted, limited to a single experimental animal, in accordance with the most recent ARRIVE animal ethical guidelines. The sample size, although small, is normal for a pilot study, with respect to the same guidelines [39]. 

## 5. Conclusions

Within the limits of the present study, it can be concluded that properly embedded and handled fluorescently un-labelled “thick” sections (250–300 µm) of non-demineralized hybrid specimens of experimentally induced peri-implantitis in dog models provide easily obtainable information. They can be efficiently examined under CLSM to reveal sufficient data about soft-tissue-bone-implant details, when compared to costly and time-consuming traditional light microscopy histological protocols. Data obtained in this way may lack the precision of LM approaches on thin sections, but can provide complementary information, like 3D models. Investigation of such specimens in CLSM may seem technologically demanding, yet the richness of data acquired may justify this approach. Future studies may focus on a risk-free manipulation of the sections, as well as on refining of the histological protocols aimed for such investigation.

## Figures and Tables

**Table 1:** Linear measurements between the landmarks on both aspects of the thin and thick sections.

Linear Measurements between Landmarks		Definition
Horizontal measurement	imp-BC	The implant surface (imp) and the alveolar bone crest (BC)
Vertical measurements	IS-BC	The implant shoulder (IS) and the level of the bone crest (BC)
	IS-BD	The implant shoulder (IS) and bottom of the bone defect (BD)
	BC-BD	The bone crest (BC) and the bottom of the bone defect (BD)
	PM-BC	The margin of the peri-implant mucosa (PM) and the level of the bone crest (BC)
	PM-IS	The margin of the peri-implant mucosa (PM) and the implant shoulder (IS)
	PM-BD	The margin of the peri-implant mucosa (PM) and bottom of the bone defect (BD)
	aPlaque-BD	The most apical extension of the submarginal biofilm that was interposed between the implant and the pocket epithelium of the peri-implant mucosa (aPlaque) and bottom of the bone defect (BD)
Angle	IS-BD BD-BC	The angle between the IS-BD and BD-BC segments

**Table 2:** Histomorphometric results: measurements performed on “thick” and “thin” sections in CLSM; for comparison, LM thin sections were used (mean values and standard deviations).

Histometric Measurements Means (SD)		CLSM, “Thick” (250–300 µm) Sections		CLSM, “Thin”(30 µm) Sections		LM, “Thin”(30 µm) Sections	
		Buccal	Lingual	Buccal	Lingual	Buccal	Lingual
Horizontal measurement (mm)	imp-BC	1.590 (1.33)	3.493 (1.34)	0.965 (0.95)	1.444 (0.64)	0.794 (0.73)	1.546 (0.78)
Vertical measurements (mm)	IS-BC	-	-	2.262 (0.95)	0.341 (0.65)	2.244 (1.11)	0.574 (0.52)
	IS-BD	-	-	2.727 (1.20)	1.652 (1.247)	2.63 (1.33)	1.82 (1.37)
	BC-BD	0.526 (0.88)	2.085 (1.05)	0.465 (0.39)	1.319 (0.80)	0.384 (0.34)	1.452 (0.87)
	PM-BC	3.597 (1.14)	1.979 (0.43)	2.305 (1.14)	1.361 (0.75)	2.46 (1.13)	1.554 (0.83)
	PM-IS	-	-	0.758 (0.92)	0.616 (1.70)	0.214 (1.08)	1.1828 (1.26)
	PM-BD	4.123 (1.47)	4.054 (0.70)	2.770 (1.01)	2.709 (1.38)	2.848 (1.12)	3.01 (1.48)
	aPlaque-BD	3.248 (0.28)	2.399 (0.62)	2.032 (0.58)	1.495 (0.23)	2.398 (0.55)	2.21 (0.46)
Angle (degrees)	IS-BD BD-BC	75.6 (16.9)	54.25 (16.13)	59.2 (8.58)	50.2 (10.59)	77 (41.99)	47 (11.13)

## Data Availability

Data are available from the corresponding author upon reasonable request.

## Supplementary Materials

The following supporting information can be downloaded at: https://www.mdpi.com/article/10.3390/jcm12072462/s1, VideoFile No. 1: 3D reconstruction of the non-ligated implant #4, on the buccal aspect; VideoFile No. 2: 3D reconstruction of the ligated implant #5, on the oral aspect; Figure 3 Comparative images of the same implant, between a thin section in light microscopy, Masson Goldner Anilin blue stain (A), same thin section in CLSM (B) and a thick section in CLSM (C).
